# PCRMLP: A Two-Stage Network for Point Cloud Registration in Urban Scenes

**DOI:** 10.3390/s23125758

**Published:** 2023-06-20

**Authors:** Jingyang Liu, Yucheng Xu, Lu Zhou, Lei Sun

**Affiliations:** 1 College of Artificial Intelligence, Nankai University, Tianjin 300071, China; 2120210419@mail.nankai.edu.cn (J.L.); zhoulu@nankai.edu.cn (L.Z.); 2 School of Informatics, University of Edinburgh, Edinburgh EH8 9YL, UK; s2038119@ed.ac.uk

**Keywords:** DBSCAN, deep learning, instance level, point clouds, registration, urban scene

## Abstract

Point cloud registration plays a crucial role in 3D mapping and localization. Urban scene point clouds pose significant challenges for registration due to their large data volume, similar scenarios, and dynamic objects. Estimating the location by instances (bulidings, traffic lights, etc.) in urban scenes is a more humanized matter. In this paper, we propose PCRMLP (point cloud registration MLP), a novel model for urban scene point cloud registration that achieves comparable registration performance to prior learning-based methods. Compared to previous works that focused on extracting features and estimating correspondence, PCRMLP estimates transformation implicitly from concrete instances. The key innovation lies in the instance-level urban scene representation method, which leverages semantic segmentation and density-based spatial clustering of applications with noise (DBSCAN) to generate instance descriptors, enabling robust feature extraction, dynamic object filtering, and logical transformation estimation. Then, a lightweight network consisting of Multilayer Perceptrons (MLPs) is employed to obtain transformation in an encoder–decoder manner. Experimental validation on the KITTI dataset demonstrates that PCRMLP achieves satisfactory coarse transformation estimates from instance descriptors within a remarkable time of 0.0028 s. With the incorporation of an ICP refinement module, our proposed method outperforms prior learning-based approaches, yielding a rotation error of 2.01° and a translation error of 1.58 m. The experimental results highlight PCRMLP’s potential for coarse registration of urban scene point clouds, thereby paving the way for its application in instance-level semantic mapping and localization.

## 1. Introduction

Point cloud registration is the task of estimating the rigid transformation that aligns a pair of overlapping point clouds. It is important for autonomous driving [1,2], pose estimation [3,4], 3D reconstruction [5,6], and simultaneous localization and mapping (SLAM) [7,8]. In particular, in the domain of autonomous driving, registering urban point clouds presents unique challenges due to their sparsity, multiple dynamic objects, and susceptibility to environmental influences during data collection. These characteristics make feature extraction and registration particularly challenging.

Common registration methods include the iterative closest point (ICP) [9] algorithm and related approaches such as generalized ICP (GICP) [10] and normal distributions transform (NDT) [11]. These methods solve the registration problem by iteratively finding the closest points and computing the optimal transformation. However, ICP often converges to local optima when dealing with non-convex problems and heavily relies on the quality of initial values. Recently, learning-based methods have become more and more popular. Methods based on deep learning can extract more robust features and establish correspondences [12,13,14] or solve transformations in an end-to-end manner [15,16]. However, when dealing with urban point clouds, prior works need to downsample the initial data, which is time consuming and makes the algorithm sensitive to sampling density and susceptible to the influence of point cloud scale. Furthermore, these methods face challenges when dealing with geometrically similar scenarios [17] and dynamic objects. As a result, point cloud registration in urban scenes remains a significant challenge.

Previous overlap-based [18] and correspondence-based [12,16] methods usually run into difficulties when being applied to urban scenes due to noise points and repeated geometric features.The relative position of static instances in the surrounding environment remains constant during vehicle movement, which makes instance-level registration a more logical approach. Moreover, instance features are more robust and computationally efficient. Prior works usually extract features or superpoints [19] with DNNs (deep neural networks), while it is more intuitive to obtain concrete instances directly. We propose to utilize abundant semantic features in urban scenes by mapping point clouds to a high-dimensional feature space, namely, an instance level. This representation method can effectively reduce data volume from hundreds of thousands of point clouds to several instance descriptors. Such a scene representation makes it easier for robots to locate through instances in the scene as humans do, which is our key motivation. The proposed network first uses an efficient point cloud semantic segmentation model to extract point-wise semantic label, then obtains instance bounding box information through the DBSCAN [20] clustering algorithm. Unlike previous feature-pair-based methods, the instance descriptors of input point clouds are fed into the network to obtain the transformation directly.

**Figure 1:**
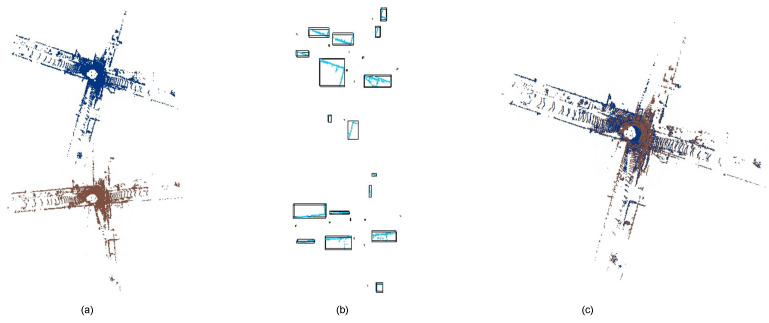
The process of PCRMLP. (**a**) Point clouds before registration, the source point cloud (red) and the target point cloud (blue). (**b**) Instance descriptors generated from semantic segmentation and DBSCAN clustering. (**c**) Point clouds registered by PCRMLP.

Overall, the paper’s main contributions can be summarized as follows: (1) Leveraging semantic segmentation and DBSCAN clustering: The paper introduces a novel approach that utilizes a semantic segmentation network and DBSCAN clustering to extract instance descriptors from urban scenes. This approach proves to be more robust compared to previous methods that relied on geometric features. The point cloud is transformed into a map consisting of instances, as depicted in Figure 1b. (2) Registration MLP network: The paper proposes a registration MLP network that directly estimates the transformation from the input instance maps. This approach is computationally efficient and avoids the need for predicting point correspondences, leading to improved performance in terms of both accuracy and speed. (3) Experimental validation: The proposed method is evaluated on a registration dataset generated from KITTI odometry. The experimental results demonstrate that the framework outperforms prior learning-based methods in terms of mean rotation error, mean translation error, and recall. This highlights the effectiveness and superiority of the proposed approach.

## 2. Related Works

In this section, the related 3D point clouds processing methods and point cloud registration methods will be briefly introduced.

### 2.1. 3D Point Clouds Processing

Existing point cloud processing algorithms can be broadly categorized into point-based methods and voxel-based methods based on the input data structure.

Point-based methods take advantage of some inherent symmetric functions (e.g., shared MLP) to handle unordered 3D point clouds. PointNet [21] is one of the pioneer attempt of point-based methods. Vanilla PointNet shows remarkable efficiency in processing irregular 3D point clouds. However, it cannot aggregate local features. Subsequent studies focus on modeling local contexts. Refs. [22,23,24] hierarchically stack the PointNet module and apply a local neighborhood query module to extract the local context.

Voxel-based methods utilize volumes to represent point clouds. Ref. [25] initially introduces 3D CNN (the convolutional neural network) to process voxelized 3D point clouds. VoxelNet [26] discards empty voxels to generate a sparse tensor to reduce the memory usage and computation cost significantly. In most cases, the higher the voxel resolution is, the better the performance is, and the more computation is required.

Several subsequent studies have aimed to combine the advantages of both point-based and voxel-based methods. One such approach is point-voxel convolution [27], which incorporates a point-branch and a voxel-branch to leverage the strengths of PointNet and sparse 3D CNN, respectively; then, the fused features will be applied to different tasks.

### 2.2. Optimization-Based Registration

The ICP algorithm is one of the most well-known registration methods, which operates in two stages iteratively: correspondence acquisition and transformation estimation. In the correspondence stage, the algorithm finds the closest points between the two point clouds being registered. In the transformation estimation stage, it solves a least squares equation to compute the optimal transformation that aligns the two point clouds.

Several implementations and variants of ICP have been proposed to improve its efficiency or accuracy and to handle specific challenges. For example, GICP introduced additional features such as surface normals or color information to enhance the accuracy of correspondence finding. Another example is the point-to-plane ICP (PL2P-ICP) [28], which incorporates plane-to-plane distances in the least squares formulation to better handle planar surfaces.

Optimization-based methods, including ICP, are mathematically rigorous and have the advantage of being able to recover closed-form solutions. They iteratively refine the transformation estimate until convergence, gradually improving the alignment between the point clouds.

### 2.3. Learning-Based Registration

The learning-based method introduces DNNs to extract more robust local or global features. These works are mainly divided into two types: learning-based feature extraction and matching methods, and end-to-end methods.

The first type utilizes DNNs as feature extractors to capture local features and corresponding relationships in point cloud scenes for following estimation via classical methods (e.g., RANSAC [29]). FCGF (the fully convolutional geometric feature network) [12] proposes a fully convolutional geometric feature network, which efficiently learns more compact geometric features. DCP (the deep closest point) [30] makes a hard assumption about the distribution of points and corresponding points and is not suitable for partially overlapping scenes.

End-to-end methods, on the other hand, employ end-to-end networks to solve the registration problem. These methods take two frames of point clouds as input and output the predicted transformation. Traditional optimization ideas are integrated into the network training process, and the loss function serves as a constraint solution [31]. PointNet [21], known for its strong neighborhood coding ability in point clouds, has been utilized in methods such as deepVCP [16] and PCRNet [15] to extract point cloud features and estimate pose transformations using MLPs. In [32], reliable line features from poles and buildings are extracted to perform registration. With the development of transformers in the computer vision domain, researchers have utilized the attention mechanism to estimate the transformation. PCAM [33] and GeoTransformer [34] apply transformers to model the correspondence between extracted local features. PREDATOR [18] proposes overlap-attention to share information between latent encodings for input point clouds.

Learning-based methods can extract more robust features and more accurate corresponding relationships from the scene for transformation estimation. These methods only use semantic information as a constraint on overlap regions or super point pairs, failing to make use of the instances in the scene to locate similar human beings. However, in urban scenes, there are a lot of noise points or repeated geometric features, which make overlap or correspondence-based methods difficult to predict the transformation.

## 3. Method

**Figure 2:**
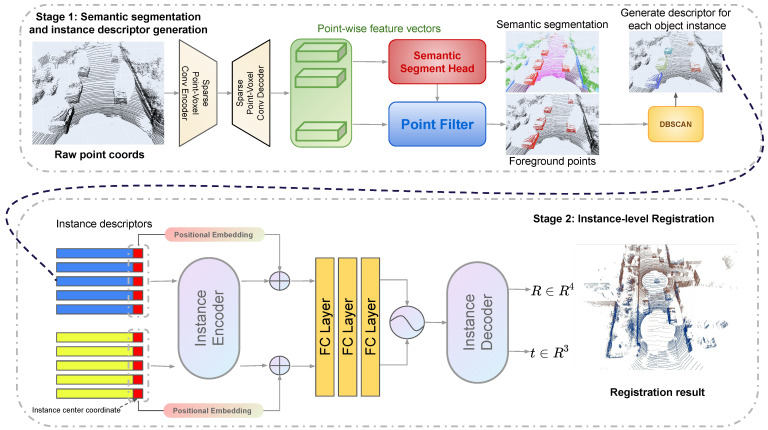
PCRMLP architecture: the model contains two stages: (1) Semantic segmentation and instance descriptor generation. We utilize our proposed object mask generator [35], which consists of the PVConv feature extract module, the 3D U-Net segmentation module, a simple task head, and DBSCAN clustering, to extract instance-wise masks of specified semantic labels. Then, the axis-aligned instance bounding boxes are obtained just via open3D. (2) Instance-level registration. We use shared MLPs in an encoder–decoder manner and estimate the transformation from input instance descriptors.

In this section, we will introduce the proposed two-stage registration framework, PCRMLP (point cloud registration MLP), which aims to estimate the transformation between a pair of raw and irregular point clouds from an urban scene. The overall structure of the PCRMLP framework is illustrated in Figure 2. It consists of two main stages: the descriptor generation stage and the registration stage with MLPs.

### 3.1. Problem Statement

Given two point clouds $P=\{p_{i}\in R^{3}\;|\;i=1,\cdots ,m\}$ and $Q=\{q_{i}\in R^{3}\;|\;i=1,\cdots ,n\}$, the goal is to recover a rigid transformation T={R,t}, the rotation matrix R∈SO(3), and the translation vector $t\in R^{3}$, which align *P* and *Q*. The transformation can be estimated by solving

(1) $$\underset{R,t}{\arg\min}\sum_{(p_{i},q_{j})\in C}∥Rp_{i}+t-q_{j}∥$$

where $p_{i},q_{j}$ are the corresponding points in the source and target point cloud.

### 3.2. Overview of PCRMLP

PCRMLP is a novel two-stage point cloud registration framework designed specifically for urban scenes, as depicted in Figure 2. The framework combines semantic segmentation and DBSCAN clustering in the first stage to generate instance descriptors and an instance map. This initial stage serves the purpose of reducing the data volume by grouping points into semantically meaningful instances, including buildings, poles, and traffic signs. By leveraging the power of semantic segmentation and DBSCAN clustering, the framework effectively identifies and labels different object instances within the point cloud data. The instance descriptors produced in the first stage are compact representations of the instances and contain essential information such as coordinates, box size, and semantic labels. These descriptors facilitate a reduction in data complexity and enable the framework to accurately locate and recognize instances in the scene, emulating the perceptual capabilities of humans as they navigate by their surroundings. In the second stage, the framework employs multi-layer perceptrons (MLPs) to estimate transformations on an instance level. Unlike traditional methods that rely on predicting correspondences between individual points in the point clouds, PCRMLP takes a direct approach by operating MLPs on the input instance maps. This innovative strategy eliminates the need for explicit correspondence estimation, leading to improved efficiency in estimating the transformations between the instance maps. By adopting a two-stage approach, PCRMLP presents a robust solution for point cloud registration in urban scenes. The framework effectively leverages instance-level information and MLPs for accurate transformation estimation, overcoming the limitations of conventional methods. The proposed framework contributes to the advancement of point cloud registration techniques in urban environments and holds great potential for various applications in robotics, autonomous navigation, and urban mapping.

### 3.3. Instance Descriptor Generation

Stage 1 of our previous work PointTrans [35] is introduced to generate instance-wise masks. Point-voxel convolution [27] is adopted to extract features from raw points. Then a 3D U-Net [36] is leveraged as the semantic segmentation branch due to its strong capacity of learning and segmentation on voxel-based representation. The following is a segmentation head that uses a simple, full connection to project the features to semantic labels. Given the semantic label of each point, the object points can directly be selected out according to its label. 3D point clouds show apparent separability in original 3D space because of their natural depth information, which means individual point cloud object instances can be segmented from the object points using simple cluster methods in 3D space. However, the number of object instances in each point cloud frame can vary in different scenes. Therefore, it is not feasible to rely on cluster methods that require the exact number of instances to be known in advance. To address this challenge, we propose the use of a density-based clustering method called DBSCAN, which is particularly well-suited for such requirements as it does not rely on knowing the exact number of instances beforehand. Instead, it groups points based on their density and spatial proximity. In the context of our framework, DBSCAN effectively segments individual instances directly from the point cloud data, as illustrated in Figure 2.

Only static instances of buildings, poles, and traffic lights are retained for further registration. To facilitate the association and recognition of the scene by a vehicle, a bounding box is generated for each filtered semantic point instance. The bounding box is solely based on the filtered semantic points and provides spatial information about the instance. Considering the importance of capturing the relationships among instances, a descriptor vector is created for each instance. This descriptor vector contains various components, including the coordinates, box size, and semantic label, which are used to implicitly learn associations between instances through a neural network. The descriptors are defined as F={coor,l,w,h,L}; $coor\in R^{3}$ is the coordinate of the center point of the generated bounding box; l,w,h are the length, width, and height of the box; and $L\in R^{3}$ is the semantic label of the instance that is embedded in a one-hot vector.

In conclusion, the 3D semantic segmentation method is adopted in the proposed network as an accurate region proposal module. We ingeniously take advantage of the separability of 3D point clouds and combine the 3D semantic segmentation method with a density-based cluster method to directly generate masks for every object instance. However, this also means that the detection performance of our algorithm will, to some extent, rely on the segmentation result of the semantic segmentation stage [35].

### 3.4. Instance-Level Registration

Considering that instance-level representation already contains rich geometric and semantic information, simple MLPs are applied to estimate the transformation from two instance-level frames. Moreover, the instance center coordinates are embedded with MLPs as positional embedding and feed the embeddings to the instance feature vectors. The positional embedding (PE) module can be defined as

(2) $$PE\left(Ins_{i}\right)=MLP\left(x_{i},y_{i},z_{i}\right)$$

where $PE(Ins_{i})$ denotes the position embedding of instance *i* and where $\left[x_{i},y_{i},z_{i}\right]$ is the coordinate of the center of the *i*th instance.

**Figure 3:**
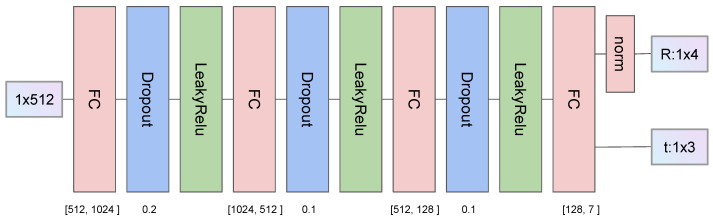
Decoder architecture. We use four full connection layers to map high-dimension features to the output transformation. The rotation is represented by quaternions, which is limited by the norm layer dividing each output by the sum of their squares. Hyperparameters are shown in the figure.

Similar to Siamese architecture, shared MLP is introduced as an encoder to map the descriptors to high dimensional space. After concatenating two feature tensors, a similar MLP decoder maps the features back to the output estimation. Figure 3 shows the decoder module. To satisfy the quaternion limitation, a normalization operation is applied in the rotation prediction branch. The output rotation is represented by quaternion since it is continuous.

### 3.5. Loss Function

As the point-wise semantic labels are only required for point filter, the semantic segmentation stage and the subsequent registration stage are trained separately.

**Semantic segmentation loss:** Following the prior point cloud semantic segmentation algorithm, we use a simple cross entropy loss function for this stage.

(3) $$L_{sem_{c}ls}=\sum CE\left(\hat{s}em_{cls},sem_{cls}\right)$$

**Registration loss:** The loss function in the registration stage restricts the predicted transformation to be as close to the ground truth as possible. We sample *n* points from the source point cloud and calculate the average distance between the virtual points projected by the estimate value and the ground truth. The loss function can be defined as

(4) $$L_{reg}=\frac{1}{n}\sum_{x\in P_{s}}∥T\left(x\right)-\hat{T}\left(x\right)∥$$

where $P_{s}$ is the source point cloud, *x* is the sampled source point, and *T* and $\hat{T}$ are the ground truth and estimation of the transformation.

## 4. Experiments and Results

In this section, the implementation details of PCRMLP will be introduced first. Then, the evaluation of PCRMLP on the Semantic KITTI dataset [37] will be illustrated. Afterwards, we demonstrate a comparison with other point cloud registration methods in terms of accuracy and computational efficiency.

### 4.1. Dataset and Training

The proposed network is trained and evaluated on the dataset generated from the Semantic Kitti odometry dataset, which contains point cloud data collected by Velodyne HDL64 LiDAR, ground truth poses provided by GPS, and point-wise semantic labels. In the KITTI point cloud dataset, sequences (0, 5, 7, and 8) are collected in urban road scenes. For each frame in these sequences, we take every third frame as its corresponding frame, with a maximum interval of 30 frames, resulting in 111,060 pairs of point clouds. We divide all point cloud pairs into a training set of 100K pairs, a validation set of 1150 pairs, and a test set of 9910 pairs. Details of the dataset are shown in Table 1. Data augmentation methods are applied, including adding random scaling ∈[0.9,1.1], random translation ∈[−0.05,0.05], and random rotation $\in [0^{\circ },360^{\circ })$ around three axis.

In the descriptor generation stage of the proposed framework, the raw points are fed to the model. In stage 1, following the structure of [36] but with a modification, conventional convolution operations are replaced with sparse point-voxel convolution [27]. This modification allows for more efficient processing of the raw point cloud data. In the next step, a semantic segmentation head is incorporated into the network to project features onto point-wise semantic labels. This semantic segmentation step helps assign meaningful semantic labels to individual points in the point cloud. Subsequently, the static object points are filtered by selecting specific semantic labels. This filtering process is performed to retain only the points corresponding to static objects of interest in the scene, such as buildings, poles, and traffic signs. To cluster the object instances, the DBSCAN algorithm is employed. The advantage of DBSCAN is that it does not require the exact number of object instances as input. Different parameters of the DBSCAN clustering algorithm are set for different classes of object instances. For building instances, *eps*, the maximum distance between two points is set as 2.3, and the minimum number of points of each neighborhood is set as 80. For pole instance, we set *eps* as 2 and set the minimum number of points of each neighborhood as 1. For traffic sign instance, the parameters are 3 and 1.

In the registration stage, the feature dimension is set as 256, the encoder and the positional embedding module MLPs are (512, 256), and the decoder MLPs are (1024, 512, 128, and 7). LeakyReLU is used as the activation function.

The models are trained and evaluated on a single RTX-TiTAN GPU and Intel Xeon Gold 5218 CPU. We conduct the experiments in python 3.7 and pytorch 1.12.1 environments. During the training period, random rotation and translation are applied to each pair of point clouds as data augmentation. We use the Adam optimizer with an initial learning rate of 1 × 10^−3^; the learning rate begins to exponentially decay with gamma of 0.99 after a warm-up period of 20 epochs.

### 4.2. Evaluation Metrics

The metrics of [14] are used to measure the performance of the proposed method on the test split. The formulas are $TE=∥\hat{t}-t_{gt}∥$ and $RE=arccos[Tr\left(R_{gt}^{T}\hat{R}-1\right)/2]$ for translation error (TE) and rotation error (RE). In addition, the recall is calculated according to the pre-set threshold, which is the success rate of registration.

### 4.3. Comparison with Existing Methods

**Figure 4:**
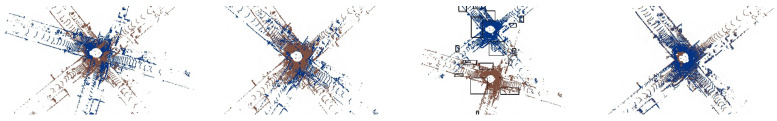
Qualitative visualization on test split. Left to right: a pair of point clouds, ICP result, PCRMLP stage 1 result, and PCRMLP registration result.

**Figure 5:**
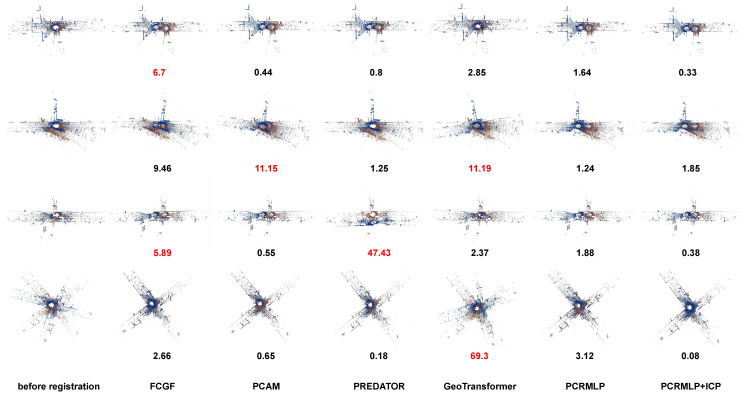
Failure cases of FCGF, PCAM, PREDATOR, and GeoTransformer. We define rotation error (RE, introduced in 4.2) > 5° as failure registration. RE in degrees is attached below each registration result.

Models are evaluated on the test split. The performance of the proposed model is compared to other methods in Table 2. We compare PCRMLP with classical ICP [9], RANSAC [29], learning-based FCGF [12], PCAM [33], PREDATOR [18], and GeoTransformer [34]. Figure 4 shows the coarse registration result from PCRMLP and fine registration result from PCRMLP combined with ICP. The proposed method with ICP outperforms on mean RE, mean TE, and recall. As discussed in [17], we notice that, as Figure 5 shows, FCGF, PCAM, and PREDATOR tend to degenerate when the scene contains lots of geometrically similar objects (e.g., cars, buildings); GeoTransformer falls into local minimal when dealing with large rotation; and the proposed method performs better in urban scenes. It is difficult for the method based on overlaps or corresponding relationships to deal with scenes that have more noise points or repeated geometric features. In these cases, the method based on instance will be more robust. PCRMLP can provide a satisfactory coarse registration initial value for ICP.

**Overfitting Discussion**: Firstly, we apply data augmentation which, is introduced in 4.1 to reduce overfitting. Secondly, the registration dataset is generated from the KITTI odometry dataset and separated to splits randomly, and the validation split is kept to validate the trained model, so that the test split is used just once. Moreover, dropout is introduced to limit the learning ability of the model. We also set the weight decay in the Adam optimizer to reduce overfitting.

### 4.4. Run-Time Analysis

The running time of PCRMLP and other methods is tested on the point cloud pairs containing 35K points. Table 3 shows that the proposed method achieves better computational efficiency. For FCGF, the running times of the feature extraction (feat) module and registration (reg) module are tested separately. Notably, the registration stage of PCRMLP only required 0.0028 seconds per pair of point clouds. This remarkable efficiency can be attributed to our approach of decomposing the dense point cloud data into a smaller set of instance data, which significantly reduces the computational burden.

## 5. Discussion

In this section, we will analyze the advantages and disadvantages of the introduced method in conjunction with prior works and discuss the direction of our future research. Most previous learning-based registration methods focus on extracting local features and correspondences to constrain rigid transformation; their performance will degrade in urban scenes with more noise points and similar geometric features, while it is more logical and humanized to recognize the scene by concrete instances such as buildings, traffic signs, and their relative relationship. Therefore, we propose the instance-level urban scene representation method to provide a novel scene recognization paradigm for autonomous vehicles. This process also effectively reduces the data volume from tens of thousands points to dozens of instances. Then, we design a simple registration network with MLPs to implicitly extract the relationship between instances. The proposed method can estimate a coarse registration from the instance-level scene with just 0.0028 s.

The observed decrease in performance of PCRMLP as the rotation increases can be attributed to two factors. Firstly, the rough estimation of the bounding boxes based on segmented points introduces errors in the instance localization. This can lead to misalignments during the registration process. Improving the accuracy of the bounding box estimation could help alleviate this issue. Secondly, MLPs have limited rotation invariance, which makes it challenging for the network to predict large rotation angles accurately. This limitation can impact the performance of PCRMLP when dealing with significant rotations between point cloud pairs. Exploring alternative network architectures or incorporating rotation-invariant modules could potentially improve the network’s ability to handle larger rotation angles. Additionally, the decrease in performance when applying the trained model to new city point cloud data highlights a limitation in generalization. The model may struggle to adapt to variations in the data distribution and scene characteristics. Fine-tuning the model on the target dataset or augmenting the training data with diverse urban scenes could help improve generalization. Despite the observed performance degradation, the initial registration results obtained from PCRMLP can still serve as valid initial values for subsequent fine registration algorithms such as ICP. These initial values provide a starting point for the iterative refinement process, which can help achieve accurate alignment between point cloud pairs.

Noticing the strong ability of reducing data volume and generating robust scene representation, we plan to apply the proposed method to the SLAM system for global localization and relocalization.

## 6. Conclusions

In this work, we propose a two-stage urban scene point cloud registration network, PCRMLP. In the first stage, we combine semantic segmentation and DBSCAN clustering to generate the instance-level scene representation, which is conducive to following registration based on instances and their relative relationships. In the second stage, simple shared MLPs are introduced to implicitly match instances between the point clouds and achieve coarse registration based on the instance-level representation. In contrast to previous learning-based works that just use semantic information as a constraint for registration, this work elevates to the instance level to perform localization in a way that is more in line with human localization habits. We train and test PCRMLP on the dataset generated from KITTI odometry. The experiment results show that the proposed PCRMLP achieves satisfactory and real-time performance on urban point clouds, with 2.01° in RE, 1.58 m in TE, and 93.24% in recall. When the rotation increases or the scene has fewer instances, the performance of PCRMLP decreases. In the future, we are going to combine instance features with geometric features to deal with scenarios that have fewer instances. We will generalize the proposed instance-level scene representation method and the registration network to instance-level semantic robot SLAM systems.

## Figures and Tables

**Table 1:** Details of dataset. In the KITTI odometry dataset, sequences 0, 5, 7, and 8 are collected in urban road scenes. For each frame in these sequences, we take every third frame as its corresponding frame, with a maximum interval of 30 frames, resulting in 111,060 pairs of point clouds. We divide the dataset into a training set of 100K pairs, a validation set of 1150 pairs, and a test set of 9910 pairs.

	Data Volume (Pairs)			
sequence	0	5	7	8
	44,310	26,510	9910	30,330
split	train	val	test	total
	100,000	1150	9910	111,060

**Table 2:** Performance comparison of rigid registration with previous methods on the *test* split set. Recall is defined as RE < 5°.

Method	RE (Deg)↓		TE (m)↓		Recall↑
	Mean	Median	Mean	Median	
ICP [9]	7.55	1.67	10.31	8.29	71.50%
RANSAC [29]	6.65	1.13	2.85	0.24	83.08%
FCGF [12]	7.88	1.55	5.97	1.56	90.35%
PCAM [33]	2.81	**0.29**	3.68	0.14	92.08%
PREDATOR [18]	3.52	0.32	3.80	**0.11**	93.13%
GeoTransformer [34]	10.56	2.21	11.04	9.72	61.10%
PCRMLP(ours)	3.42	1.56	3.56	2.77	81.82%
PCRMLP+ICP(ours)	**2.01**	0.79	**1.58**	1.03	**93.24%**

The upward arrow denotes that a higher value of the evaluation index indicates better performance of the model, while the downward arrow signifies the opposite. To highlight the best value for each evaluation indicator, we have emphasized it using bold formatting.

**Table 3:** Running times of ICP, RANSAC, FCGF, PCAM, PREDATOR, and PCRMLP on point cloud pairs containing about 35K points. We also tested the running time of stage 2 of PCRMLP, which indicates the potential of the introduced instance-level representation method.

	ICP	RANSAC	PCAM	Predator	FCGF		Ours		
					Feat	Reg	PCRMLP	PCRMLP + ICP	Stage 2
time (s)	10.31	7.64	**0.42**	1.11	0.05	12.05	0.65	4.01	**0.0028**

## Data Availability

The data generation code will be available on request.
